# No more monkeying around: primate malaria model systems are key to understanding *Plasmodium vivax* liver-stage biology, hypnozoites, and relapses

**DOI:** 10.3389/fmicb.2015.00145

**Published:** 2015-03-26

**Authors:** Chester Joyner, John W. Barnwell, Mary R. Galinski

**Affiliations:** ^1^Malaria Host–Pathogen Interaction Center, Emory Vaccine Center, Yerkes National Primate Research Center, Emory UniversityAtlanta, GA, USA; ^2^Malaria Branch, Division of Parasitic Diseases and Malaria, Centers for Disease Control and PreventionAtlanta, GA, USA; ^3^Division of Infectious Diseases, Department of Medicine, Emory UniversityAtlanta, GA, USA

**Keywords:** malaria, *Plasmodium*, *P. vivax*, *P. cynomolgi*, liver, hypnozoite, dormancy, non-human primate animal models

## Abstract

*Plasmodium vivax* is a human malaria parasite responsible for significant morbidity worldwide and potentially death. This parasite possesses formidable liver-stage biology that involves the formation of dormant parasites known as hypnozoites. Hypnozoites are capable of activating weeks, months, or years after a primary blood-stage infection causing relapsing bouts of illness. Elimination of this dormant parasitic reservoir will be critical for global malaria eradication. Although hypnozoites were first discovered in 1982, few advancements have been made to understand their composition and biology. Until recently, *in vitro* models did not exist to study these forms and studying them from human *ex vivo* samples was virtually impossible. Today, non-human primate (NHP) models and modern systems biology approaches are poised as tools to enable the in-depth study of *P. vivax* liver-stage biology, including hypnozoites and relapses. NHP liver-stage model systems for *P. vivax* and the related simian malaria species *P. cynomolgi* are discussed along with perspectives regarding metabolite biomarker discovery, putative roles of extracellular vesicles, and relapse immunobiology.

Malaria is responsible for significant morbidity, mortality, and socioeconomic hardships in about 100 countries (World Health Organization, 2014). The causative agents of the disease are parasitic protists of the genus *Plasmodium*, which have a complex life-cycle involving a vertebrate and invertebrate host. After infecting susceptible mammals, the parasite undergoes obligate, clinically silent development in the liver prior to entering the blood and causing the clinical symptoms and pathology associated with malaria. Neutralizing the parasites in the liver has been a goal to prevent blood-stage infection and, therefore, clinical disease and transmission. Indeed, targeting liver stage forms (LSFs) has been a strong theme in current anti-malarial drug and vaccine efforts (Abdulla et al., 2011; Duffy et al., 2012). Moreover, preventing relapse infections is especially important in light of research demonstrating that the majority of *Plasmodium vivax* malaria episodes are due to relapses, which result from the activation of dormant forms in the liver, and not from new, mosquito-borne infections (Betuela et al., 2012; White et al., 2014).

Naturally, human clinical studies relating to the biology of LSFs and host–pathogen interactions in the liver are prohibitive, and regardless, influenced by uncontrollable variables; e.g., diet and medications. Unlike human studies, experimental NHP model systems are well suited for studying LSFs and relapse biology. Future malaria research with non-human primates (NHPs) on these topics will undoubtedly include large-scale ‘omics,’ advanced immune profiling, mathematical modeling, computational biology, and the integration of clinical and ‘omics’ datasets (Galinski et al., 2013, 2014; Voit, 2013).

While research using NHPs will inevitably remain limited worldwide within a few capable biomedical research centers, such investigations with the public release of datasets will enable many more investigators to participate in associated areas of research and development. Moreover, collaborations with investigators at these centers can lead to new research directions including much needed translational studies to improve diagnostics and clinical care and to develop and test new anti-malarial interventions and vaccine candidates. From our perspective, these factors make NHP model systems critical to advancing the world toward malaria eradication.

## NHP-MALARIA MODELS OVERVIEW

Non-human primate model systems have been instrumental in malaria research for decades whether for furthering basic understanding of *Plasmodium* biology, malaria pathogenesis, or preclinical investigations pertinent to developing new interventions (Coatney et al., 1971; Collins, 1974; Galinski and Barnwell, 2012; Beignon et al., 2014). Notably, they were critical for the discovery of dormant forms of the parasite in the liver, known as hypnozoites. These forms were first discovered in rhesus macaques (*Macaca mulatta*) experimentally infected with *P. cynomolgi* (Krotoski et al., 1982b), and then in chimpanzees infected with *P. vivax* (Krotoski et al., 1982a). Research from the field has confirmed that hypnozoites can stay dormant for weeks, months, or years after a primary infection and then activate and result in relapses, with new cycles of blood-stage parasitemias and illness (White and Imwong, 2012). Recently, NHPs were critical for the development of an *in vitro*, primary hepatocyte culture system that supported the cultivation of *P. cynomolgi* LSFs for approximately 40 days and provided the first tangible evidence that hypnozoites existed and were capable of activating and multiplying to generate merozoites (Barnwell and Galinski, 2014; Dembele et al., 2014).

Various NHP-simian and human malaria parasite combinations can be used to study *Plasmodium* biology. Many strains of the parasites that infect NHPs, including four validated relapsing species, are available and can be used to address scientific questions relating to LSF biology (**Table 1**). Different strains of *P. cynomolgi* and *P. vivax* that possess distinctive relapse patterns can be utilized to study the consequences of frequent versus infrequent relapses on the host immune system. A suitable mouse model is not currently available to study such phenomena. Indeed, humanized mice containing human hepatocytes have been demonstrated to support *P. falciparum* liver-stage growth (Vaughan et al., 2012; Kaushansky et al., 2014). These models also appear to have some utility for *P. vivax* because they appear to support the development of hypnozoites (Mikolajczak et al., 2013). However, these mice lack intact immune systems and, thus, are deficient when addressing immunobiological questions, whether for *P. falciparum* or other primate malaria species (Kaushansky et al., 2014).

**Table 1:** *Plasmodium* species and strains with critical characteristics for studying liver-stages.

Characteristics of each species and strain									
***Plasmodium* species (strains)**	**Isolated from**	**Principal NHP host(s)**	**Primary cycle (days)**	**Relapses (yes/no)**	**Time between relapses**	**Reference**
*Plasmodium falciparum* (Salvador I or Santa Lucia)	Human	*Aotus griseimembra, A. vociferans*, *A. nancymaae*	5.5 – 6.5	No	NA	Fairley (1947); Shortt et al. (1949), Collins et al. (1977)
*P. malariae* (Uganda I)	Human	*Aotus* sp. and *Saimiri* sp.	14 – 15	No	NA	Chin et al. (1965); Lupascu et al. (1967), Collins et al. (1975)
*P. vivax* (Brazil VII)	Human	*Aotus* sp. and *Saimiri* sp.	7 – 8	Yes	Early and frequent (1 to 2 months)	Fairley (1947), Collins and Barnwell (unpublished)
*P. vivax* (Chesson)	Human	*Aotus* sp. and *Saimiri* sp.	7 – 8	Yes	Early and frequent (1 month)	Fairley (1947); Ungureanu et al. (1976), Collins et al. (1980)
*P. vivax* (Salvador I)	Human	*Aotus* and *Saimiri* sp.	7 – 8	Yes	Infrequent (2 to 4 months)	Fairley (1947); Contacos et al. (1972), Collins et al. (1973)
*P. vivax* (North Korea)	Human	*Aotus* and *Saimiri* sp.	7 – 8	Yes	Late (6 to 12 months)	Fairley (1947); Shute et al. (1976)
*P. cynomolgi* (B, M, Berok, Ceylon)	*Macaca fascicularis* and *M. sinica*	*M. mulatta*	8 – 10	Yes	Early and frequent (1 to 2 months)	Garnham (1966); Coatney et al. (1971)
*P. simiovale*	*M. sinica*	*M. mulatta*	>12 (uncertain)	Yes	Varied: frequent (2 to 4 weeks) to infrequent (few to many over 2 years)	Coatney et al. (1971); Collins and Contacos (1974)
*P. fieldi*	*M. nemestrina* and *M. fascicularis*	*M. mulatta*	>12 (uncertain)	Yes	Varied: frequent (2 to 4 weeks) to infrequent (few to many over 1 year)	Held et al. (1967); Coatney et al. (1971)
*P. knowlesi*	*M. fascicularis* and *M. nemestrina*	*M. mulatta* and *M. fascicularis*	5	No	NA	Garnham et al. (1957); Coatney et al. (1971)
*P. coatneyi*	*M. fascicularis*	*M. mulatta*	10	No	NA	Coatney et al. (1971)

### VIVAX MALARIA – NHP MODELS

While NHP models have been used occasionally to supplement fundamental *P. falciparum* research findings from culture systems and for pre-clinical studies, *P. vivax* research over the last few decades would have been virtually impossible without NHP models; i.e., *Aotus* and *Saimiri* monkeys (Galinski and Barnwell, 2012). Unlike *P. falciparum*, a long-term *in vitro* culture system for *P. vivax* does not currently exist due to the need for a regular supply of reticulocytes (Noulin et al., 2013), and thus, we believe yet to be defined culture media components that better mimic the host environment are also needed (unpublished data). In the meantime, NHP models have been critical for generating *P. vivax* material for in-depth analyses (Anderson et al., 2015) and NHP experimental studies continue to complement and expand upon blood-stage analyses that are now possible with small clinical samples attained from human infections (Russell et al., 2012; Galinski et al., 2014).

**FIGURE 1:**
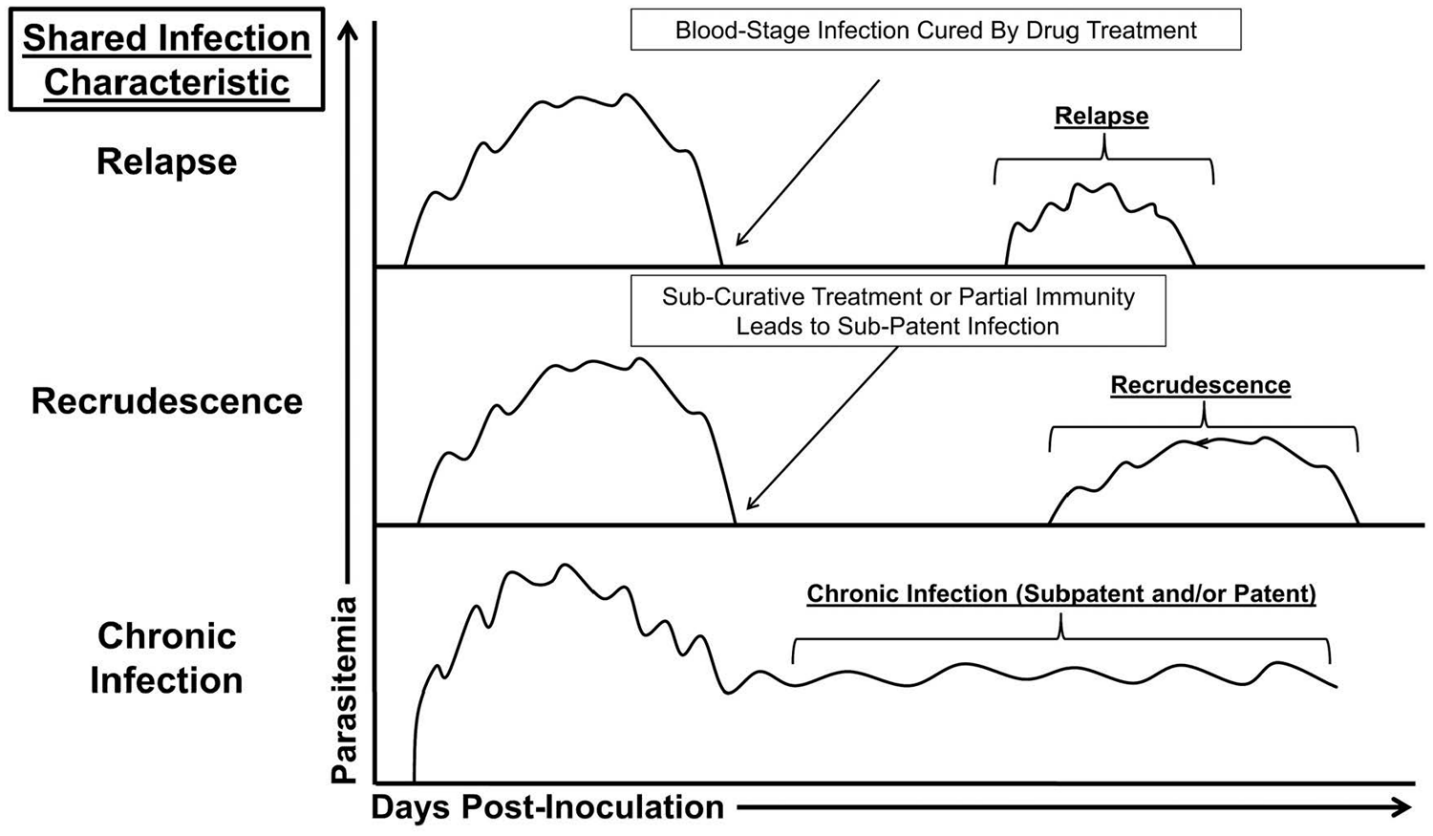
**Shared infection characteristics of human and simian malaria parasites.**.

To investigate hypnozoites and relapses, *Aotus* or *Saimiri* species can be infected with NHP-adapted *P. vivax* strains via mosquito inoculation or syringe injection of sporozoites into a blood vessel (**Table 1**; Galinski and Barnwell, 2012; Galinski et al., 2013). Similar to human infections, relapsing, recrudescing, or chronic infection profiles can be observed in these models provided the animals are splenectomized to interfere with an overly robust removal of infected erythrocytes (**Figure 1**). In contrast to relapses, recrudescences are the result of untreated or persistent blood-stage infections that become sub-patent, below the level of detection by microscopy, followed by an eventual return to patency; such recurring parasitemias are distinct from relapse parasitemias that are due to the activation of hypnozoites and release of a new brood of merozoites from the liver.

Blood-stage parasitemias, which begin to develop within 8–10 days, can be curatively treated without destroying the hypnozoites. PCR testing can confirm the absence of blood-stage parasites, and thus, any subsequent blood-stage infections can be confirmed as relapses and not recrudescences. This experimental strategy is currently the only reliable means to study vivax relapses, with the caveats that these animals are small (typically about 1 kg), parasitemia is typically low or moderate (1–2%), and only small blood volumes can be taken (6 ml/kg/month) based on Institutional Animal Care and Use Committee (IACUC) guidelines. One strain in particular (named the Brazil VII strain) is being developed at the Centers for Disease Control and Prevention (CDC) for studying relapses as it shows multiple relapse patterns over a period of several months similar to that observed previously in humans with “tropical strains” (**Table 1**).

### SIMIAN PARASITE – NHP MODELS FOR VIVAX MALARIA

Simian malaria parasite-NHP models are powerful systems to investigate LSF biology, hypnozoites, and relapses compared to the small New World NHPs. Simian malaria parasites productively infect Old World monkeys, including rhesus macaques (*M. mulatta*) and long-tailed macaques (*M. fascicularis*), which possess similar genetic composition and physiology to humans (Gardner and Luciw, 2008; Messaoudi et al., 2011; Zimin et al., 2014). The macaques are much larger than New World monkeys, which allows for greater blood or bone marrow draws (up to a maximum of 10 ml/kg/month) for isolation of parasite material and host cells for immunobiological studies. Additionally, more reagents exist for experimentation with these NHP species. Furthermore, large amounts of liver-material can be collected via biopsies or whole livers to isolate LSFs for downstream experiments.

*Plasmodium cynomolgi* is a “sister species” of *P. vivax.* These closely related parasites share similar biology such as the formation of hypnozoites and caveolae vesicle complexes in infected erythrocytes (Aikawa et al., 1975; Akinyi et al., 2012; Tachibana et al., 2012). Tens of millions of *P. cynomolgi* sporozoites can be generated in a specified, experimental time-frame compared to *P. vivax* sporozoites which are more difficult to generate (Rosenberg and Rungsiwongse, 1991; unpublished data). They can then be used to experimentally infect macaques and lead to productive blood-stage parasitemias (Collins et al., 1999). Furthermore, the same experimental approach used in Small New World Monkey infections with *P. vivax* can be employed to study relapses (see Vivax Malaria – NHP Models). Two other simian malaria species that could be useful for studying hypnozoites or relapse mechanisms are *P. fieldi* and, especially, *P. simiovale* (**Table 1**).

As with *P. vivax*, many *P. cynomolgi* isolates exist that have their own infection and relapse characteristics (**Table 1**). The number and frequency of relapses in rhesus infected with different strains of *P. cynomolgi* can be predicted with greater accuracy than typically possible with New World monkey-adapted *P. vivax* isolates although *P. vivax* Brazil VII has so far been uniquely dependable in this regard (unpublished data). In rhesus, variables such as the sporozoite inoculum can be altered to produce consistent infection patterns even though inter-individual variability between animals as well as inherent properties of the strain being used in the study can influence infection kinetics (Schmidt, 1986).

*Plasmodium cynomolgi* can be genetically manipulated more easily than *P. vivax,* though *P. vivax* transfection has been accomplished at the CDC and Emory, where transient transfection of trophozoites was demonstrated in *Saimiri boliviensis* (Pfahler et al., 2006). In recent years, we have developed *P. cynomolgi* parasites with integrated transgenes, including a *red fluorescent protein* (*rfp*) gene (Akinyi et al., 2012; unpublished data). Transient RFP- and green fluorescent protein-expressing *P. cynomolgi* parasites have also been reported and used to purify *P. cynomolgi* LSFs from NHP primary hepatocyte cultures using fluorescence-activated cell sorting (Voorberg-van der Wel et al., 2013). Technical hurdles such as achieving better yields of purified parasites for downstream experiments still remain. Nonetheless, these are monumental breakthroughs given the challenges working with these parasites.

### PRIMARY NHP HEPATOCYTE CULTURES

NHP primary hepatocyte cell cultures are valuable for exploratory studies and may prove to be crucial for validating *in vivo* experiments as well as mechanistic studies requiring gene knockdowns, drug treatment, etc. Critical advancements have been made toward optimizing *in vitro* studies with *Plasmodium* LSFs, particularly with *P. cynomolgi* and rhesus monkey primary hepatocytes, even though infection rates, and thus, parasite yields still remain low (Voorberg-van der Wel et al., 2013). The most critical advancement has been the establishment of a long-term culture system using primary NHP hepatocytes that can support *P. cynomolgi* hypnozoites and other LSFs for up to 40 days; importantly, this system allows for hypnozoites to activate and develop into schizonts capable of releasing merozoites (Barnwell and Galinski, 2014; Dembele et al., 2014). This breakthrough ramps up research in this area and provides a workable system for validating *in vivo* findings.

## SYSTEMS BIOLOGY APPROACHES

Systems biology approaches have been recently pioneered to study infectious diseases and vaccine efficacy (Li et al., 2014; Petrizzo et al., 2014; Zak et al., 2014). Systems biology foregoes traditional reductionist approaches and focuses on a biological system in its entirety. Typically, multiple ‘omics technologies are employed to generate large datasets and methods are developed to integrate those datasets. Advanced mathematics and statistics are required to generate computational models of specific biological processes, such as hematopoiesis, immune responses, and infectious disease pathogenesis. Indeed, these strategies are being utilized by the Malaria Host–Pathogen Interaction Center (MaHPIC) using Old World and New World monkey models to study malaria and bring a wealth of data and novel results to the research community via online resources. We believe that systems biology approaches will likewise have utility for studying *Plasmodium* LSFs and the mechanisms behind relapses.

Systems biology methods can be attempted to study LSFs using transgenic, fluorescent LSFs isolated by fluorescence-activated cell sorting from *ex vivo* liver tissue. We and others (Voorberg-van der Wel et al., 2013) hope to demonstrate that adequate, purified *ex vivo* parasite material for downstream experimentation can be attained using such strategies. Performing transcriptomic, proteomic, and metabolomic analyses on the *ex vivo* material holds potential for identifying biochemical and molecular pathways important in hypnozoite biology. A hypnozoite proteome may help elucidate new vaccine candidates whereas transcriptome and metabolome data could give insight into other potential biochemical and molecular pathways that could become drug targets. Indeed, headway is being made in understanding hypnozoite biology with the recent demonstration that epigenetic programming could be responsible for latency (Dembele et al., 2014). Notably, however, modifying epigenetic changes *in vivo* may have detrimental side effects to the host organism, and thus, this may not be a feasible treatment strategy in humans.

We are well aware of the scientific and physiological challenges, but also optimistic that highly sensitive systems biology approaches that include metabolomics can help identify biomarkers that could predict the presence of hypnozoites and serve as diagnostic tools. Metabolomics is a relatively new, yet powerful, scientific discipline that is gaining traction in the fight against malaria (Olszewski et al., 2009; Kafsack and Llinás, 2010; Lakshmanan et al., 2011; Salinas et al., 2014). It is exciting to consider how high-resolution mass spectrometry could potentially identify a metabolic biomarker(s) (predictably of host origin) in the serum, plasma, urine, or saliva that is indicative of the presence of LSFs and the need for treatment. Potential biomarkers are best sought from an *in vivo* infection where host–parasite interactions can be investigated in the context of the normal physiology of the parasite and the host. Experimental NHP models can be informative in this regard. Diets and other variables can be controlled, and infected samples can be collected daily, or even multiple times daily if useful, over the course of a designated infection period through multiple relapse episodes without the immediate, ethical need for treatment.

## EXTRACELLULAR VESICLES: POTENTIAL DIAGNOSTIC AND THERAPEUTIC TARGETS

Extracellular vesicles (EVs) are a heterogeneous population of small vesicles found in virtually all bodily fluids including serum, plasma, urine, saliva, etc., and are categorized into subtypes based on their physical properties such as density, size, and shape as well as their biogenesis (Kowal et al., 2014; Robbins and Morelli, 2014). These vesicles are produced by all multi- and unicellular organisms examined to date, and different types of EVs contain specific protein and RNA cargo dependent upon the cell-type the EV originated from (Villarroya-Beltri et al., 2014).

The roles of liver-derived EVs on liver physiology have been reviewed elsewhere (Imani Fooladi and Mahmoodzadeh Hosseini, 2014). Notably, multiple studies have implicated liver-derived EVs in the life-cycle of liver pathogens. For example, exosomes, a specific EV subtype that originates from a multivesicular body within the cell, derived from liver non-parenchymal cells were demonstrated to transfer resistance against hepatitis B virus infection to hepatocytes via an IFN-α mediated mechanism (Li et al., 2013). Despite these implications of EVs in the liver, putative roles of EVs have not been reported with regards to *Plasmodium* liver-stage biology, but warrant attention. Indeed, EVs were recently shown to be released from *Plasmodium* infected erythrocytes and implicated in a “density-dependent sensing mechanism” that influences *P. falciparum* gametocytogenesis (Mantel et al., 2013; Regev-Rudzki et al., 2013). These studies were the first to raise the possibility for a natural role of EVs during the life-cycle and provide a firm rationale that such mechanisms may exist for LSFs.

Extracellular vesicles could predictably serve as a communication mechanism between LSFs and influence hypnozoite activation, and NHP, primary hepatocyte cultures can be used to assess if such mechanisms exist. Cultures can be infected with sporozoites, and EVs derived from uninfected and infected hepatocytes can be isolated from the culture medium (Momen-Heravi et al., 2013). The EVs can then be placed on LSF cultures harboring different forms of the parasites, including hypnozoites, to directly test if EVs isolated at different points of the LSF cycle (e.g., when hypnozoites are activating) result in specific biological outcomes. For example, one may predict that EVs isolated from cultures with activated hypnozoites may signal other hypnozoites to exit dormancy and multiply. These experiments are not perfect, however, and will be unable to delineate if the effect is caused by host and/or parasite-derived EVs, but it is a starting point to explore mechanisms and specific EV components that could potentially be used to force hypnozoite activation.

If such a strategy of ‘waking up’ hypnozoites is feasible, akin to the novel ideas suggested by others based on drug interventions to target putative epigenetic control mechanisms of dormancy (Dembele et al., 2014), only the blood-stages would have to be treated, making treatment more straightforward with multiple safe options compared to primaquine with its contraindications (White et al., 2014). Indeed, as members of the MaHPIC, we have been developing EV research strategies utilizing NHPs infected with simian malaria parasites. EVs are challenging to purify, but we have managed to purify them from small volumes of plasma, observe them by electron microscopy, and confirm their identity through other biochemical and physical means (unpublished data).

Hepatocyte-derived EVs in the circulation could also comprise possible biomarkers of hypnozoites similar to biochemical biomarkers identified by metabolomics (Deng et al., 2009; Vlassov et al., 2012). Hepatocytes and other tissue-resident cells in the liver release EVs that contain specific protein and RNA cargo that become altered during liver injury (Farid et al., 2012). Similarly, we hypothesize that the microRNA and protein content of liver-derived EVs will be altered when hepatocytes are infected with *Plasmodium*. If this proves to be the case, EVs could be potentially isolated from serum, plasma, saliva, and/or urine and specific diagnostic tests developed to use EVs as diagnostic markers of hypnozoites. Although it may seem unlikely that EVs from *Plasmodium*-infected hepatocytes could serve as biomarkers because of the scarcity of infected hepatocytes (a relative few in an entire liver), EVs are currently being explored to develop sensitive methods to detect cancer in bodily fluids (Rak, 2013). If EV-based diagnostic approaches are feasible and promising for cancers where few malignant cells exist, such approaches are worth exploring for developing technologies to detect hypnozoites.

## RELAPSE IMMUNOBIOLOGY

The immunobiology behind relapses, recrudescences, and chronic infections (**Figure 1**) during malaria is poorly understood although “immune exhaustion” during chronic infections has been investigated recently (Wykes et al., 2014). This neglected area of research needs attention because each of these distinctive infection profiles could have unique effects on the host immune response; e.g., to alter the host’s memory pool.

The team of immunologists at the MaHPIC has been using NHP models to understand the effects of relapses on the host immune system. Indeed, the consortium has determined that relapses cause continuous expansion of the circulating memory B-cell pool using the *P. cynomolgi*-rhesus model system (unpublished data). Currently, the team is performing follow-up experiments to better understand this phenomenon and its immunological relevance using sampling strategies that are not possible in humans. Blood is being collected before, during, and after relapses to monitor the alterations in the B-cell compartment by flow cytometry, with a special interest in memory B cells. The identities of predominant B-cell clones based on immunoglobulin sequences are being examined using Ig-Seq technologies (Georgiou et al., 2014). The first goal is to determine the clonal diversity of the B-cell recall response against relapsing or challenge parasites. The second goal is to assess which B-cell clones respond during consecutive blood-stage infections. If consecutive blood-stage infections are selecting for a particular subset of B-cells or, contrastingly, inducing proliferation of different B-cells, the host’s memory B-cell pool could be significantly altered. We predict that alterations of the B-cell compartment could translate into poor recall responses because memory B-cells, critical for detecting, expanding, and producing antibodies to eliminate the parasites, might be eliminated from the memory B-cell niche by other B-cells that predominantly proliferate. If this proves to be the case, the impact must be considered in light of developing a vaccine that relies on neutralizing antibodies mediated by memory B-cells.

## CONCLUSION

Non-human primate models of malaria have enabled major contributions toward understanding liver-stage biology, hypnozoites, and relapses, and will continue to provide the means to investigate this enigmatic part of the *Plasmodium* life-cycle. Primaquine is the only FDA-approved drug against hypnozoites despite its contraindications. Additionally, excessive use of this drug can support the rise of primaquine resistant parasites (John et al., 2012; Price, 2014). A biomarker test would help restrict treatment to only those individuals in need, and be useful in malaria elimination campaigns where it is preferable to only treat infected individuals instead of everyone to ensure elimination of the parasite reservoir. New knowledge, techniques, and possible diagnostics, vaccines, and medications that may result from studying LSFs using NHP models will inevitably be key to malaria eradication efforts.

## AUTHOR CONTRIBUTIONS

All authors contributed to the writing, figure and table, and reviewed and approved the finalized article.

## Conflict of Interest Statement

The authors declare that the research was conducted in the absence of any commercial or financial relationships that could be construed as a potential conflict of interest.
